# Effect of temporary weaning and creep feeding on calf growth and the reproductive efficiency of their Hereford dams

**DOI:** 10.5713/ab.21.0384

**Published:** 2022-03-02

**Authors:** R. Santa Cruz, I. De Barbieri, V. Morales Olmos, F. Montossi, C. Viñoles

**Affiliations:** 1 Meat and wool Production national research program, INIA Tacuarembó, Route 5 km 386, 45000, Tacuarembó, Uruguay; 2 Polo Agroforestal, Estación Experimental Bernardo Rosengurtt, Facultad de Agronomía, Universidad de la República, Route 26 km 408, 37000, Cerro Largo, Uruguay; 3 Centro Universitario Tacuarembó, UdelaR, 45000, Uruguay

**Keywords:** Beef Cow Efficiency, Cattle Farming, Early Conception, Pregnancy Rate, Weaning Weight

## Abstract

**Objective:**

The objective was to test if creep feeding (CF) improves the average daily gain (ADG) and weaning weight of calves submitted to temporary weaning (TW) and if the combination of CF and TW improves conception and pregnancy rates of cows.

**Methods:**

Primiparous (n = 74) and primiparous and multiparous (n = 104) cows grazing native grasslands were used in experiment 1 and 2; respectively. The experimental design was in plots divided into complete random blocks with two replications. The CF was the big plot and TW the small plot, thus four experimental groups were formed: i) −CF−TW (n = 21 and 27); ii) −CF+TW (n = 16 and 24); iii) +CF−TW (n = 20 and 26); iv) +CF+TW (n = 17 and 27) with cow-calf pairs for experiments 1 and 2; respectively. Nose plate application for TW had a duration of 14 and 15 days for experiment 1 and 2: respectively. In experiment 1, calves were fed at 1% of live weight for 112 days using a commercial supplement with 18.4% crude protein. In experiment 2, the supplementation lasted 98 days, and was carried out with corn dried distillers grains with soluble (DDGS) at 40% of the potential intake on a daily basis.

**Results:**

The TW reduced ADG during the TW period and the following 14 days, but the negative effect of TW was maintained until the final weaning only in experiment 2. The CF increased ADG during TW period in both experiments. The TW promoted an earlier conception of the dams (12 days in −CF treatment and 19 days in +CF treatment, p<0.01) and CF increased pregnancy rate in experiment 1, being the effects not consistent between experiments.

**Conclusion:**

The CF consistently promoted an increase in ADG during the period of TW and increased final weaning weight of calves, therefore it is economically viable.

## INTRODUCTION

Beef cow efficiency is influenced by the reproductive performance and the weight of calf weaned per breeding female [1]. The combined effects of calf suckling and nutrition are the major factors influencing the resumption of postpartum ovarian cycles [2]. Continuous calf suckling blocks ovulation with the consequent longer period of postpartum anestrus, contributing to a reduced reproductive efficiency [3]. Beef cows grazing *Campos* natural grasslands [4] are exposed to seasonal changes in the quantity and quality of available forage. The greatest variability of summer coincides with the breeding season and the pre-weaning period [5]. Thus, interventions that increase the availability of nutrients for the cow-calf pair before weaning would enhance the productivity of the beef herd.

Temporary weaning (TW) avoids suckling, reduces milk production and generates a positive metabolic signal promoting a redistribution of nutrients in the cow [6]. When TW is correctly applied, an increase in pregnancy rate and a reduction in the calving – conception interval is achieved [7]. The response to TW is dependent on the interaction between body condition and the metabolic status of the dams, with a recommended stable condition [7] score of 3.5 (scale 1 to 8, [8]). However, TW decreases average daily gain (ADG) of calves during the treatment period and for up two weeks after the removal of the nose plates. The decrease is associated with the absence of milk in the diet, digestive disorders when restarting the dairy diet and/or a reduction in cow’s milk production [6]. As a consequence, TW can result in lighter calves by the time of definitive weaning [9].

Offering supplementary feed to lactating calves, using a creep feeding (CF) system, could improve the energy balance of the cow [10] and weaning weight of the calves [11]. CF improves the energy balance of the cows through a greater availability of forage [10], since it is not related to a decrease in the suckling frequency of the calves [11]. There are a large number of reports on the positive effect of CF on the live weight of calves, suggesting that supplementation is an efficient management alternative during the phase of rapid growth [12]. The economic viability of CF depends on the cost of the supplement, the price of the calf and the feed conversion efficiency [13]. Considering the advantages and disadvantages of TW and CF, their combination appears as an interesting alternative to increase the productivity of the beef herd.

Therefore, the current study was designed to test the hypothesis that CF allows improvement in the ADG of calves to which TW is applied and thus avoiding the loss of weight at weaning. We also tested if CF associated with TW advances the moment of conception and improves pregnancy rate of cows. The objective of this study was to test the impact of feeding calves a supplement associated or not to TW, on their ADG and weaning weight and the reproductive efficiency or their dams.

## MATERIALS AND METHODS

The studies were designed according to the recommendations set by the Uruguayan Honorary Committee for Animal Ethics and all applied protocols were approved by INIA Animal Ethics Committee, approval N° INIA_2013.1(experiment 1) and INIA_2014.31b (experiment 2).

### Animals and location

Two experiments were carried out at Glencoe Experimental Unit of the National Institute for Agriculture Research of Uruguay (INIA) (32°00′21″ S and 57°08′06″ W). Experiment 1 started on 15th November 2012 until 21st March 2013, and experiment 2 from 9th of December 2015 until 18th of March 2016. In experiment 1, 74 primiparous calved cows with an initial live weight of 418±55.3 kg and an average body condition of 4.1±0.47 in a scale 1 to 8 [8] were used. The average live weight and age of the calves at the beginning of the experiment were 83±17.3 kg and 75±13.0 days, respectively. In experiment 2, 104 (29 primiparous and 75 multiparous) calved cows were used. The initial live weight and body condition were 501±46.6 kg and 4.8±0.70 (scale 1 to 8), respectively. The average live weight and age of the calves at the beginning of the experiment were 109±17.5 kg and 80±14.9 days, respectively. The mating period lasted 60 and 62 days for experiment 1 and 2, respectively. A reproductive evaluated bull to every thirty cows was used. Each week, bulls were rotated between paddocks to avoid genetics effect.

### Experiment design and treatments

**Figure 1:**
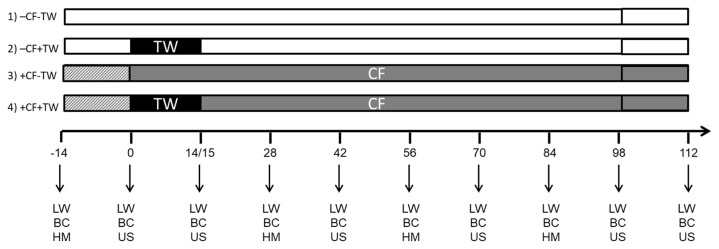
Schematic representation of the experimental design with the groups with (+) or without (−) creep feeding (CF) and with (+) or without (−) temporary weaning (TW): 1) −CF−TW, 2) −CF+TW, 3) +CF−TW, 4) +CF+TW. TW had a duration of 14 and 15 days and CF 112 and 98 days for Experiments 1 and 2, respectively. The target amount of supplement was gradually increased during a 10 day period, before the application of TW (Day 0). Live weight (LW) and body condition score (BC) were measured every 14 days. Herbage mass (HM) was evaluated at the beginning of the experiment and every 28 days. Ultrasounds (US) were performed at the start and end of the TW and from there every 28 days until the end of the experiment.

The experiment design was in plots divided into complete random blocks with two replications in each experiment. The big plot was defined by the supplementation, with (+) and without (−) CF, forming two groups of animals in two plots. While the small plot was defined by the application of TW (with + and without −), remaining both groups grazing together. Thus, four experimental groups divided in two plots were formed: i) −CF−TW (n = 21 and 27); ii) −CF+TW (n = 16 and 24); iii) +CF−TW (n = 20 and 27); 4) +CF+TW (n = 17 and 27) for cow-calf pairs in experiment 1 and 2, respectively (Figure 1). In both experiments the beginning of TW was defined as day 0 of the experimental design.

### Temporary weaning

For experiment 1, TW commenced at the same time of the beginning of mating and lasted for 14 days. For experiment 2, TW commenced 14 days after the beginning of mating and had a duration of 15 days (Figure 1). Plastic nose plates for suckling restriction were applied to the calves while remaining with their dams. The nose plates were placed in half of the calves of each experimental group. Treatments were blocked by age (primiparous and multiparous in experiment 2), live weight and body condition of the cows and the date of birth and sex of the calves.

### Creep feeding

Calves were first exposed to the supplement in the cattle pen, and the adaptation period included a progressive increase in the amount of supplement offered (0.2% of live weight every 2 days) until reaching the desired amount. In both experiments, this period started 10 days before the application of TW. Calves had unrestricted access to the supplement in an area designed to prevent access of their dams. In experiment 1, calves were fed at 1% of the live weight until day 112 using a commercial supplement. In experiment 2, the supplementation lasted 98 days using corn dried distillers grains with solubles (DDGS), a by-product of the ethanol industry. Metabolizable energy (ME) and crude protein (CP) requirements of lactating calves were obtained from the literature [14]. Based on milk production (7 litres) and composition from previous trials using the same herd, total inputs (ME 20.3 MJ/d and CP 245 g/d) would allow for an ADG of 0.945 kg/d, considering that milk is the first election of calves [15]. Three levels of DDGS in the diet (20%, 30%, and 40%) were simulated to reach the target ADG of 1.200 kg/d, based on the total CP and ME supply [14]. The inclusion of 40% DDGS in the diet resulted the best option, being 100% the potential intake of these calves. The amount of supplement was adjusted every 2 weeks, according to the live weight change of the calves.

#### Composition of supplements

In experiment 1, the supplement used in the CF pens was a commercial formulation with the following ingredients: Corn, wheat, soy flour, whole soybeans, sunflower flour, barley, gluten feed, rice bran, wheat bran, molasses, whey milk powder, calcium carbonate, bicalcium phosphate, salt, premix of vitamins and minerals (Erro Nutrición Animal, Soriano, Uruguay). In experiment 2, the supplement used was corn DDGS. The DDGS analysis provided information on total CP and the acid detergent insoluble nitrogen, to estimate the quantity of nitrogen available for ruminal or intestinal degradation (Table 1) [16]. The chemical composition for both supplements analysed in the Animal Nutrition Laboratory of INIA La Estanzuela are presented in Table 1.

### Forage mass

Forage mass was evaluated 2 weeks before the beginning of the experiment (day -14), and from day 0 every 4 weeks (days 28, 56 and 84; Figure 1) using the comparative yield method described by Haydock and Shaw [17]. For each treatment, five reference quadrats (scale 1 = minimum to 5 = maximum forage available) of 0.125 m^2^ and their replicates were cut at ground level. Thereafter, the paddock was walked in zigzag to evaluate visually 100 quadrats per paddock using the points of the defined scale. The wet and the dry weight (60°C until constant weight) of each sample was recorded. A linear regression equation between the scale points and their dry matter (DM) content, allowed estimating the amount of kg DM/ha in the paddock, according to the frequency of each scale point recorded [17]. Using this information, the monthly evolution of forage allowance was estimated (kg DM/kg live weight per ha) [18].

### Animal measurements

#### Live weight and body condition

All animals were weighed unfasted at the beginning of the experiment and every 14 days until the end of the experimental period (Figure 1). Weights were always recorded in the morning, to minimize the effect of filling. The group order and the scale (True-test GR 3000; Muñoz y Arquero, Montevideo, Uruguay) were the same for all determinations. ADG between live weight measurements was calculated, dividing the kg gained by the days between each measurement.

Coinciding with the live weight measurement (Figure 1), the body condition of all the cows was determined by visual appreciation by the same trained observer, using the method described by Vizcarra et al [8], scale of 1 to 8 (1 = emaciated animal and 8 = animal with excess of fat).

#### Pregnancy diagnosis and embryo/fetal age

The diagnosis of gestation and the age of the embryos/fetuses were determined by ultrasonography. The ultrasounds were performed using an Agroscan ultrasound machine with a dual linear transducer of 5.0/7.5 MHz for transrectal use (Biotay SA, Montevideo, Uruguay) on days 0, 14, 42, 70, 98, and 112 for experiment 1, and on days 0, 15, 42, 70, and 98 for experiment 2 (Figure 1). Using gestational age, the date of conception was calculated (date of pregnancy diagnosis – gestational age) to define the day of conception from the start of the mating [19].

### Economical evaluation

A marginal analysis of the gross income (difference between marginal income and marginal cost) for both treatments was carried out, considering only the costs and income in addition to a net income that already exists. Then, marginal income was calculated as calf price (USD/kg)×(weight gained per day with supplement [kg] – weight gained per day without supplement [kg])×number of days of the treatment. The marginal cost was calculated as freight cost of the supplement plus supplement cost× plus labor costs for additional management. For the calculations, some assumptions were made, such as the price of the calf (2.05 USD/kg of calves with a weight greater than 180 kg, and 2.30 USD/kg of calves with a weight less than 180 kg), the cost of infrastructure (5.7 USD per calf) and extra labor costs due to the supplementation (6 USD per calf).

### Statistical analysis

All variables for experiments 1 and 2 were analysed independently.

#### Forage measures

Continuous variables (forage mass and forage allowance) were analysed using the MIXED procedure of SAS (version 9.4, SAS Institute Inc., Cary, NC, USA). The model included the fixed effects of the big plot (+CF and −CF), observation and their interaction. The random effect was the replication. Differences between means were considered significant if p<0.05. The means were presented as least square means±standard error.

#### Animal measures

Continuous variables (live weight of calves and cows, body condition of cows, ADG of calves), adjusting live weight in cows and calves and body condition in cows by their initial determination, were analysed using the MIXED procedure of SAS. The distributions of the residuals were evaluated using the univariate procedure and extreme data was eliminated. Comparisons between groups were made by analysis of variance. The model included the fixed effects TW, CF, their interaction, observation (obs), and the triple interaction TW×CF×obs. The random effect was the plot, and the interaction plot×TW nested with the plot. The categorical variable (pregnant/not pregnant) and the moment of conception (interval from start of mating to conception) were analysed using the GENMOD procedure and survival test of SAS, respectively. Differences between means were considered significant if p<0.05 and trends were identified when 0.05<p≤0.10, adjusted by the Tukey-Krammer test. The means were presented as least square means±standard error.

## RESULTS

### Forage measures

No significant differences were observed in forage mass and forage allowance between −CF and +CF groups during both experimental periods (p>0.05; Table 2).

### Animal measures

No significant interaction between the fixed effects (TW and CF) was observed for any of the variables modelled in both experiments (p>0.05).

#### Daily weight gain of the calves

**Figure 2:**
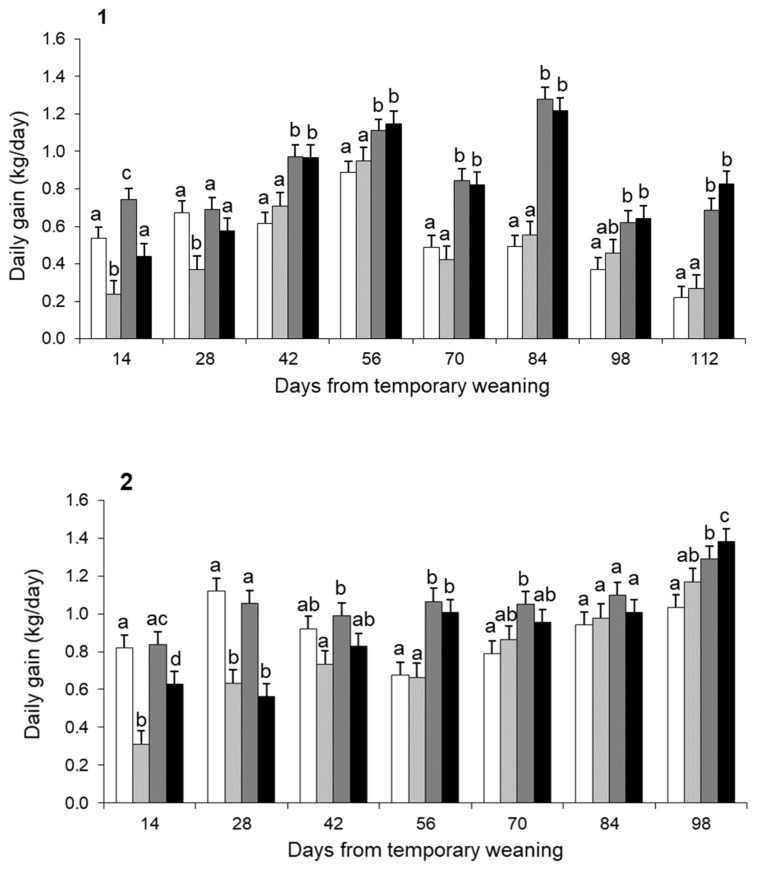
Evolution of daily weight gain of calves during the experimental period for groups with (+) or without (−) creep feeding (CF) and with (+) or without (−) temporary weaning (TW). −CF−TW (white bar); −CF+TW (light grey bar); +CF−TW (dark grey bar); +CF+TW (black bar) groups for Experiment 1 (1) and Experiment 2 (2). ^a–d^ Treatments with different letters within the same observation are statistically different (p<0.05).

Temporary weaning reduced the ADG of calves during the period it was applied and fourteen days later (day 28) in both experiments (Figure 2A, 2B). In experiment 1, the daily weight gain of +TW calves was 44% of the −TW calves by the time the nose plates were removed (day 14) (p<0.01), and 55% fourteen days later (p<0.01; Figure 2A). Similarly, in experiment 2, +TW calves gained 38% and 56% of the −TW calves during the period the nose plates were in place (p<0.01) and fourteen days later (p<0.01; Figure 2B). A residual effect of TW on average daily weight gains up to weaning was observed only in experiment 2 (−TW = 0.977±0.0166 kg vs +TW 0.837±0.0170 kg; p<0.01).

Creep feeding affected the ADG of calves during the en tire period in experiments 1 (+CF = 0.816±0.018 vs −CF = 0.516±0.018 kg/d p<0.01) and 2 (+CF = 0.982±0.017 vs −CF = 0.832±0.017 kg/d p<0.01). Moreover, CF increased the ADG during the TW period by 202% in experiment 1 (p< 0.05) and 186% in experiment 2 (p<0.01; Figure 2A, 2B).

#### Live weight of the calves

**Figure 3:**
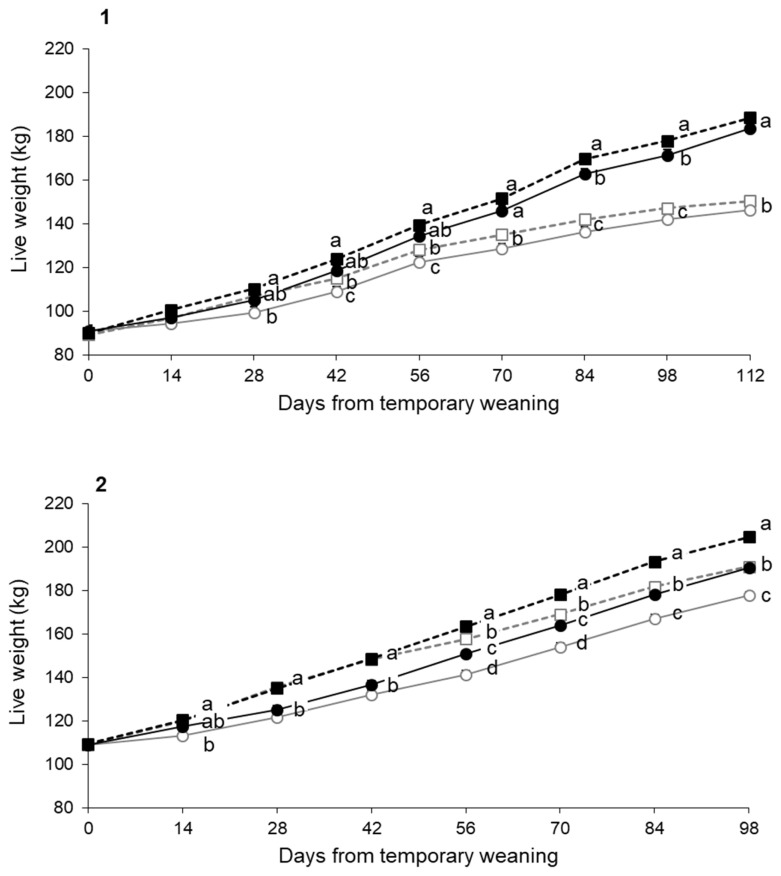
Evolution of live weight of calves from temporary weaning (day 0) until final weaning (day 112 for experiment 1 and 98 for experiment 2) for groups with (+) or without (−) creep feeding (CF) and with (+) or without (−) temporary weaning (TW). −CF−TW (white square and grey dotted line); −CF+TW (white circle and grey continuous line); +CF−TW (black square and black dotted line); +CF+TW (black circle and black continuous lines) groups for Experiment 1 (1) and Experiment 2 (2). ^a–d^ Treatments with different letters within the same observation are statistically different (p<0.05).

The evolution of the live weight of the calves was affected by CF and TW (Figure 3). For both experiments, the effect of CF on live weight began to be evident 2 months after the beginning of the supplementation (Figure 3).

The negative effect of TW on live weight was maintained until definitive weaning only in experiment 2 (Figure 3B). In this experiment, CF allowed to recover the weight losses associated to the application of TW (−CF−TW = 190.9±1.64 kg vs +CF+TW = 190.5±1.63 kg; p = 0.85).

#### Conversion efficiency and gross income

Despite DDGS presented lower cost than the commercial supplement (265 vs 345 USD per ton), produced lower gross income considering only calf variables (Table 3).

#### Daily Weight Gain, live weight, and body condition of the dams

In experiment 1, there was no effect of CF or TW on ADG, live weight and body condition of the dams at any time during the experimental period (p>0.05). In experiment 2, CF decreased the mean body condition of the dams (−CF = 5.10±0.040 units vs +CF = 4.96±0.038 units; p<0.05) but there was no effect of CF or TW on live weight and daily weight gain of the dams (p>0.05).

#### Day of conception and final pregnancy

**Figure 4:**
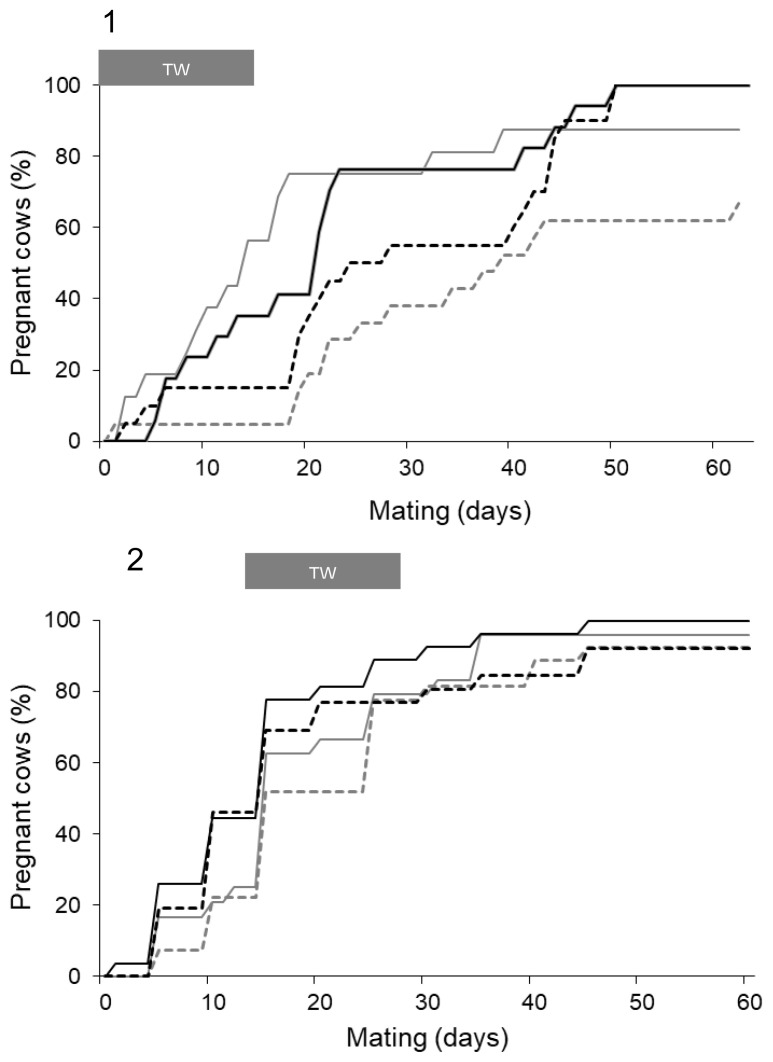
Accumulated pregnancy percentage in cows from the start of mating for groups with (+) or without (−) creep feeding (CF) and with (+) or without (−) temporary weaning (TW). TW period is indicated in the grey bar on the top. −CF−TW (grey dotted line), −CF+TW (grey continuous line), +CF−TW (black dotted line), +CF+TW (black continuous line) groups for Experiment 1 (1) and 2 (2). Grey bar in the top indicates the period of TW.

In experiment 1, TW promoted an earlier conception by the dams in +CF and −CF groups (−CF+TW = 16±3.1 days and +CF+TW = 21±3.5 days) compared to those whose calves where not submitted to TW (CF−TW = 28±3.5 days and +CF−TW = 40±4.4 days; p<0.01). As observed in Figure 4A, CF increased pregnancy rate. The group −CF−TW showed the lower pregnancy rate (67.7%) at the end of the mating period compared to −CF+TW (87.5%), +CF−TW (100%), and +CF+TW (100%); p<0.05. In experiment 2, there was no effect of CF or TW on the day of conception and pregnancy rate (p>0.05; Figure 4B).

## DISCUSSION

The hypothesis that CF allows improvement of the ADG of calves to which TW is applied and avoids the loss of weight at the final weaning was accepted. The CF improved calf ADG during the period the nose plates were placed and increased their live weight at definitive weaning. However, the hypothesis that CF associated to TW would allow advancement of the day of conception and increase the pregnancy rate of the dams was partially accepted. The CF and TW enabled advanced conception and increased pregnancy only in experiment 1, but not in experiment 2.

The negative effect of TW on ADG of calves during nose plate application and the subsequent measurement (14 days post TW) was consistent in both experiments, as was previously reported [6]. In experiment 1, calves were able to recover the live weight at definitive weaning. Cow’s body condition and high forage allowance (greater than 3.31 kg DM/kg live weight [18]) for the cow-calf unit would allow recovery of milk production quickly, recovering ADG of calves and live weight at weaning [6]. In experiment 2, however, the lower weights of TW calves were maintained until definitive weaning, regardless of the good body condition of the dams and the high forage allowance. Comparisons between experiments cannot be made, as in experiment 2 there were not enough numbers of cows in each age group, but it would be interesting to test under the same conditions, whether the lack of an effect of TW on the definitive weaning weight was related with the age of the dams (primiparous vs multiparous). Differences in the milk production curve between these categories could be part of the explanation [20]. Since the lactation curve of primiparous cows is flatter than that of multiparous cows [21], it would explain why 5 days older calves in experiment 2 weighed 26 kg more at the beginning of the experiment. Thus, the calves of multiparous cows at the time of TW were having access to a higher milk production, so the negative effect of TW would have been greater. These results are supported by previous findings [6], which reveal a hidden cost of TW and highlights the relevance of applying it in restricted conditions and in combination with alternatives such as CF. The interaction between TW and CF was not significant, probably because the duration of both treatments was different. However, the combination of TW and CF, allowed supplemented calves with TW to duplicate their ADG during that period, and avoided lower weights at definitive weaning, an effect that would be more relevant in calves born to multiparous cows. We are not aware of previous studies that combined TW and CF, but the results of the two experiments are consistent and suggest the feasibility of using CF to increase ADG during TW.

CF had a positive effect in the ADG and consequently in weaning weight in both experiments using two different supplements. Previous work also found these positive results [11,12]. The improvement in ADG of CF calves even with forage allocations that were not limiting for the cow-calf pair [18], shows that the nutrient intake and the expression of the genetic potential of calves grazing native grasslands on Basaltic soils are limited [11]. Even if calves had the capacity to select forage of greater quality, as it occurs in lambs, the CP and fibre content of the forage during summer is limiting for optimum growth [22]. Moreover, in female calves it has been suggested that there is a pre-weaning nutritional programming window that affects the onset of puberty [23,24], causing endocrinal changes associated to greater reproductive efficiency [25,26]. Therefore, calf supplementation is recommended in both the short and long-term if the technique is economic viable.

Considering only the effects of CF up to weaning, its application was economically viable in both experiments. However, using a by-product, with a lower cost in experiment 2, resulted in a lower income, due to the smaller impact of DDGS on ADG and weaning weights. An alternative to increase the economic margins of both supplements would be to feed them only around the period of TW, reducing the amount of supplement needed [12]. Using CF for a shorter period (e.g., 38 days including 10 days before TW to 14 days after), only to avoid TW losses, must be evaluated considering potential negative effects associated to the decrease in protein/energy content of the diet and the change in the diet when the supplement is removed.

There are evidences that calves prefer milk, then supplement, and finally forage [27], and this explains why the demand for milk does not decrease while consuming the supplement [28]. If CF causes changes in the energy balance of the dams, they should be explained by variations in forage availability or by a greater demand of milk by a larger calf. However, in a previous experiment, milk production assessed by the weight-suckle-weight method, was similar between cows whose calves received or not CF, even the calves being heavier [11]. We have not found differences in forage availability between treatments and CF had no impact on live weight of the dams. There was an effect only on body condition in experiment 2. In this case, multiparous cows were used, and the decrease in BC of those dams whose calves received CF, may be due to a more attached bond between multiparous cows and their calves. This behaviour makes that multiparous cows stay longer waiting for their calves while they are eating the supplement, thus reducing their own grazing sessions [20,29]. Moreover, previous reports showed that CF could have an additive effect, since the provision of a solid diet to ruminants in the suckling phase, accelerates the ruminal development and this stimulates the consumption of forage [30]. However, in these experiments we did not find differences in forage availability and evaluation of forage consumption was not performed to support this concept.

Reproductive efficiency in multiparous cows (experiment 2) in a situation of high forage allowance was not affected. This lack of an effect has been previously reported [11]. However, in primiparous cows with good body condition, CF stimulated an increase on pregnancy rate that was not associated to changes in weight or body condition. Also, as was previously reported [31], a shortening of the calving conception interval was observed in experiment 1 in cows whose calves were TW. Both tools, CF and TW, proved to have positive effects on first calf cows. Although our design does not allow us to compare between age groups, primiparous rather than multiparous cows seems to benefit more from the application of both techniques. Probably as primiparous cows are a category with greater requirements as they are still growing, both techniques may have a greater impact on their energy balance. It would be of interest to evaluate both technologies under the same conditions to be able to compare age groups.

## CONCLUSION

We concluded that CF consistently promotes an increase in ADG of the calves during the period of TW application, overriding its negative effect on live weight at definitive weaning. Furthermore, CF was an economically viable alternative in both years, but with a different gross margin depending on the type of supplement. Although TW advanced the day of pregnancy and CF increased pregnancy rate, the effects of these technologies on the dams were less consistent and requires further studies.

## Figures and Tables

**Table 1:** Chemical composition for the commercial formulation supplement used in experiment 1 and for the corn DDGS used in experiment 2

Supplement	Commercial formulation Experiment 1	DDGS Experiment 2
Acid detergent fiber (%)	17.4	22.8
Acid detergent insoluble nitrogen (%)	-	41
Metabolizable energy (ME/kg DM)	2.73	2.56
Crude protein (%)	18.4	22.6^1)^

DDGS, dried distillers grains with soluble; ME, metabolizable energy; DM, dry matter.

1) Crude protein available [16].

**Table 2:** Mean and standard error (SE) of forage variables by experiment (1 and 2) and treatment with (+) or without (−) creep feeding (CF) and with (+) or without (−) temporary weaning (TW)

Experiment	Big plot	Treatment		SE	p-value

		−CF	+CF		

	Small plot	−TW/+TW	−TW/+TW		
1	Forage mass (kg DM/ha)^1)^	2,628	2,713	172.6	ns
	Forage allowance (kg DM/kg LW)^2)^	6.6	6.4	0.46	ns
	Crude protein content (% DM)	7.3	8.1	0.73	ns
	Acid detergent fiber (% DM)	47.7	46.9	1.18	ns
2	Forage mass (kg DM/ha)^1)^	3,680	3,328	263.9	ns
	Forage allowance (kg DM/kg LW)^2)^	9.3	7.5	0.94	ns
	Crude protein content (% DM)	6.0	6.1	0.23	ns
	Acid detergent fiber (% DM)	33.0	32.9	0.24	ns

CF, creep feeding; TW, temporary weaning; SE, standard error; DM, dry matter; LW, live weight.

1) Total dry weight of forage per unit area of land (hectare).

2) Relationship between forage mass and animal live weight per hectare [4].

ns: p>0.05.

**Table 3:** Parameters related to the efficiency of supplement used, for Experiment 1 (commercial supplement 18% CP) and Experiment 2 (corn distiller’s dried grains with soluble) for each treatment, with creep feeding (+CF) and without temporary weaning (−TW), and +CF and with TW (+TW)

Experiment	Variables	Treatment	

		+CF−TW	+CF+TW
1	Mean supplement consumption (kg/d)	1.1	1.1
	Extra live weight at weaning (kg)^1)^	38.0	37.5
	Conversion efficiency^2)^	3.24	3.29
	Marginal cost (USD/calf)	49.6	49.6
	Marginal income (USD/calf)	69.8	67.7
	Extra gross income (USD/calf)	18.3	16.3
2	Mean supplement consumption (kg/d)	0.8	0.8
	Extra live weight at weaning (kg)^1)^	13.8	12.8
	Conversion efficiency^2)^	5.56	5.99
	Marginal cost (USD/calf)	26.9	26.9
	Marginal income (USD/calf)	31.5	29.6
	Extra gross income (USD/calf)	4.5	2.7

1) Difference in live weight at weaning between supplemented and non-supplemented calves.

2) Kg of supplement eaten per extra live weight kg.

## References

[1] Burns BM, Fordyce G, Holroyd RG (2010). A review of factors that impact on the capacity of beef cattle females to conceive, maintain a pregnancy and wean a calf-Implications for reproductive efficiency in northern Australia. Anim Reprod Sci.

[2] Montiel F, Ahuja C (2005). Body condition and suckling as factors influencing the duration of postpartum anestrus in cattle: a review. Anim Reprod Sci.

[3] Wettemann RP Precalving nutrition/birth weight interaction and rebreeding efficiency.

[4] Allen VG, Batello C, Berretta EJ (2011). An international terminology for grazing lands and grazing animals. Grass Forage Sci.

[5] Do Carmo M, Sollenberger LE, Carriquiry M, Soca P (2018). Controlling herbage allowance and selection of cow genotype improve cow-calf productivity in Campos grasslands. Prof Anim Sci.

[6] Quintans G, Banchero G, Carriquiry M, López-Mazz C, Baldi F (2010). Effect of body condition and suckling restriction with and without presence of the calf on cow and calf performance. Anim Prod Sci.

[7] Nava De (1994). The G effects of restricted suckling and prepartum nutritional level on reproductive performance of primiparous crossbred beef cows.

[8] Vizcarra JA, Ibañez W, Orcasberro R (1986). Repeatability and reproducibility of two scales to estimate the body condition of Hereford cows. Investig Agronómicas.

[9] Quintans G, Vázquez AI, Weigel KA (2009). Effect of suckling restriction with nose plates and premature weaning on postpartum anestrous interval in primiparous cows under range conditions. Anim Reprod Sci.

[10] Cremin JD, Faulkner DB, Merchen NR, Fahey GC, Fernando RL, Willms CL (1991). Digestion criteria in nursing beef calves supplemented with limited levels of protein and energy. J Anim Sci.

[11] Viñoles C, Jaurena M, De Barbieri I, Do Carmo M, Montossi F (2013). Effect of creep feeding and stocking rate on the productivity of beef cattle grazing grasslands. NZ J Agric Res.

[12] Carvalho VV, Paulino MF, Detmann E (2019). A meta-analysis of the effects of creep-feeding supplementation on performance and nutritional characteristics by beef calves grazing on tropical pastures. Livest Sci.

[13] Aguiar AD, Vendramini JMB, Arthington JD (2015). Limited creep-feeding supplementation effects on performance of beef cows and calves grazing limpograss pastures. Livest Sci.

[14] NRC (2001). Minerals Nutrient requirements of dairy cattle: seventh revised edition.

[15] Gutiérrez V, Espasandin AC, Astessiano AL, Casal A, López-Mazz C, Carriquiry M (2013). Calf foetal and early life nutrition on grazing conditions: metabolic and endocrine profiles and body composition during the growing phase. J Anim Physiol Anim Nutr (Berl).

[16] Mieres J, ADIN and ICP (1996). What are they, what do they measure and how are they calculated?. Hoja Divulg No 40 INIA.

[17] Haydock KP, Shaw NH (1975). The comparative yield method for estimating dry matter yield of pasture. Aust J Exp Agric Anim Husb.

[18] Sollenberger LE, Moore JE, Allen VG, Pedreira CGS (2005). Reporting forage allowance in grazing experiments. Crop Sci.

[19] Brownlie TS, Morton JM, McDougall S (2016). Accuracy of fetal age estimates using transrectal ultrasonography for predicting calving dates in dairy cows in seasonally calving herds in New Zealand. NZ Vet J.

[20] Ungerfeld R, Hötzel MJ, Scarsi a, Quintans G (2011). Behavioral and physiological changes in early-weaned multiparous and primiparous beef cows. Animal.

[21] Miller N, Delbecchi L, Petitclerc D, Wagner GF, Talbot BG, Lacasse P (2006). Effect of stage of lactation and parity on mammary gland cell renewal. J Dairy Sci.

[22] Ramos Z, De Barbieri I, van Lier E, Montossi F (2019). Body and wool growth of lambs grazing on native pastures can be improved with energy and protein supplementation. Small Rumin Res.

[23] Allen CC, Alves BRC, Li X (2012). Gene expression in the arcuate nucleus of heifers is affected by controlled intake of high- and low-concentrate diets. J Anim Sci.

[24] Gasser CL, Behlke EJ, Grum DE, Day ML (2006). Effect of timing of feeding a high-concentrate diet on growth and attainment of puberty in early-weaned heifers. J Anim Sci.

[25] Guggeri D, Meikle A, Carriquiry M, De Barbieri I, Montossi F, Viñoles C (2018). Long-term effect of early nutrition on endocrine parameters and liver and endometrial gene expression of the members of the somatotrophic axis in Hereford heifers. Reprod Domest Anim.

[26] Guggeri D, Meikle A, Carriquiry M, Montossi F, De Barbieri I, Viñoles C (2014). Effect of different management systems on growth, endocrine parameters and puberty in Hereford female calves grazing Campos grassland. Livest Sci.

[27] Lopes SA, Paulino MF, Detmann E (2016). Does supplementation of beef calves by creep feeding systems influence milk production and body condition of the dams?. Trop Anim Health Prod.

[28] Carvalho VV (2018). Preweaning nutritional effects of supplements on performance of suckling beef calves grazing tropical pastures.

[29] Le Neindre P, D’Hour P (1988). Effects of a postpartum separation on maternal responses in primiparous and multiparous cows. Anim Behav.

[30] Baldwin RL, McLeod KR, Klotz JL, Heitmann RN (2004). Rumen development, intestinal growth and hepatic metabolism in the pre- and postweaning ruminant. J Dairy Sci.

[31] Soca P, Rodríguez M, Olivera J, Villegas N, Claramunt M Effect of Short-Term Energy Supplementation and Temporary Weaning on Follicular Size and Early Pregnancy in Primiparous Cows in Anestrus.

